# Association Between the Need to Change Initial Antifungal Therapy and Treatment Costs in Patients With Invasive Aspergillosis

**DOI:** 10.1093/ofid/ofae747

**Published:** 2024-12-20

**Authors:** Barbara D Alexander, Melissa Johnson, Mark Bresnik, Vamshi Ruthwik Anupindi, Lia Pizzicato, Mitchell DeKoven, Belinda Lovelace, Craig I Coleman

**Affiliations:** Infectious Diseases Division, Duke University, Durham, North Carolina, USA; Infectious Diseases Division, Duke University, Durham, North Carolina, USA; F2G, Inc., Princeton, New Jersey, USA; IQVIA, Falls Church, Virginia, USA; IQVIA, Falls Church, Virginia, USA; IQVIA, Falls Church, Virginia, USA; F2G, Inc., Princeton, New Jersey, USA; Department of Pharmacy Practice, School of Pharmacy, University of Connecticut, Storrs, Connecticut, USA

**Keywords:** amphotericin B, antifungal agents, aspergillosis, azole, costs and costs analysis, therapy change

## Abstract

**Background:**

Early antifungal initiation in invasive aspergillosis (IA) is recommended. Changing antifungals occurs for a myriad of reasons but associated costs are unclear.

**Methods:**

US claims data for adults admitted for IA were identified from 10/1/2015 to 11/30/2022. Patients were stratified by those who did and did not change antifungal therapy. Adjusted all-cause healthcare utilization and costs/patient during index hospitalization and at 1, 6, and 12-months after the index date between the cohorts that did and did not change antifungal therapy were compared.

**Results:**

Among 1,192 IA patients, 707 (59.3%) changed their initial antifungal therapy over follow-up. The index hospital length of stay was longer (Δ = 6 days, *P* < .001) and costs were higher (Δ = $65,149, *P* < .001) in the change vs. no change cohort. Median 1, 6, and 12-months all-cause costs were higher in patients changing antifungal therapy vs. not (Δ = $90,938−$192,953).

**Conclusions:**

Changing antifungals was associated with longer hospital stays and costs and higher all-cause costs over 12-months.

## Teaser

- Nearly 60% of patients admitted with invasive aspergillosis changed their initial antifungal therapy, many within 1 to 2 weeks of initiation.
- Median index all-cause hospitalization costs were higher in the change cohort (Δ = $65 149), as were all-cause treatment costs over the next year (Δ = $192 953).

Invasive aspergillosis (IA) is a serious mold infection caused by *Aspergillus* species [1]. This opportunistic infection primarily affects individuals with weakened immune systems and is often associated with life-threatening complications as the fungus invades tissue at the primary site of infection (typically the lungs) and spreads to other organs. IA has been designated by the World Health Organization as 1 of 4 critical priority fungal pathogens, and it accounts for nearly 9000 hospitalizations annually in the United States [2, 3].

According to treatment guidelines [4, 5], early initiation of antifungal therapy in patients with or suspected of having IA is strongly recommended. Voriconazole is often the first-line treatment of choice in patients with IA, as it offers superior effectiveness and fewer adverse effects as compared with amphotericin B deoxycholate. Alternatives such as isavuconazole, posaconazole, itraconazole, and lipid formulations of amphotericin B can be employed for patients with contraindications or intolerance to voriconazole. In severe or refractory cases, combination therapy, such as voriconazole with an echinocandin, may be considered.

Costs associated with treating IA are substantial and may result from intensive diagnostic testing, specialized care, supportive treatments to manage complications, and costly antifungal therapy [1, 6]. At the time of data collection (2015–2022), some of the antifungals were generic and may have presented less of an access and affordability challenge to patients than those that remained patented with higher insurance copays or coinsurance costs. Additional reasons for changing antifungals may include switching therapy at hospital discharge to match patients’ insurance coverage and reduce out-of-pocket costs. Changing antifungal therapy due to treatment failure, adverse events, tolerability, or drug-drug interactions is common [7, 8], but few real-world data have evaluated the association between needing to change antifungal therapy and IA treatment costs.

We compared health care resource utilization and all-cause total costs for the initial hospitalization and subsequent inpatient and outpatient care for up to 12 months in patients with IA who did and did not change their initial antifungal therapy.

## METHODS

This retrospective observational cohort study utilized data from IQVIA's New Data Warehouse from January 2010 to December 2022 [9, 10]. This data set includes deterministically linked data from professional fee and prescription claims data and their hospital charge data master database from facilities across the United States. These data contain (1) ∼1 billion professional fee claims per year (representing >870 000 practitioners per month) with diagnoses, procedures, and office-administered drugs; (2) ∼1.6 billion retail or mail-order prescription claims representing dispensed prescriptions for about 85% of all pharmacies; and (3) records from >450 hospitals, including inpatient and outpatient encounters within a facility with drug, procedure, diagnosis, and charge data for the entire stay, along with patient demographics.

The data were accessed in compliance with the Health Insurance Portability and Accountability Act. Institutional review board approval was not required for this retrospective analysis of deidentified secondary data. The data utilized are available only by license through IQVIA.

Adult patients admitted for IA (index date) were identified by *ICD-10-CM* diagnosis codes (B44.0, B44.1, B44.7) in any position during the patient selection period of October 2015 to November 2022 (data prior to October 2015 were used only to assess patient baseline characteristics). The first admission for IA during the patient selection period was used. Patients with ≥1 inpatient hospitalization claim in the charge data master with a diagnosis code for IA in any position during the all-available baseline period (1 January 2010 to index date − 1) were excluded to capture patients’ first IA events. The admission date for IA was defined as the index date (ie, time 0). Moreover, patients had to (1) link to the professional fees and pharmacy claims data during the study period, (2) have evidence of any systemic antifungal therapy for ≥3 days (any combinations of azoles, polyenes, or echinocandins) during the hospitalization, (3) have activity denoted as ≥1 medical claim in the professional fee data set and ≥1 pharmacy claim in the pharmacy claim data set during the 6-month baseline period, and (4) exhibit pharmacy stability (ie, consistent reporting of data from the pharmacy most frequently visited by the patient during each month of the baseline period). Exclusion criteria included evidence of invasive fungal infections other than aspergillosis during the index hospitalization.

Patients were stratified into 2 cohorts: those who did and did not change their initial antifungal therapy. Antifungal therapies were identified by National Drug Codes, the Healthcare Common Procedure Coding System, and hospital billing descriptions and included amphotericin B (deoxycholate or lipid formulations), azoles (fluconazole, isavuconazole, itraconazole, posaconazole, voriconazole), and echinocandins (micafungin, caspofungin, anidulafungin), either alone or as combination therapy. Antifungal use was captured as part of patients’ hospital charges when administered as an inpatient and when a pharmacy claim was adjudicated in the outpatient setting. Change in antifungal therapy was defined as the initiation of a new antifungal with or without discontinuing the first antifungal therapy and could occur during an inpatient stay or as an outpatient. This would include discontinuation, switch, or modification defined as (1) failure to refill an antifungal therapy within 2 times the days’ supply of therapy with restart of a different antifungal therapy, (2) starting a new antifungal therapy within 30 days before or after discontinuation of prior antifungal therapy, or (3) the addition or subtraction of antifungal therapy from the regimen at any point during the post-index period. Those who did not change antifungal therapy were defined as those who maintained their initial antifungal therapy, discontinued antifungal therapy without restart (ended treatment), or discontinued and restarted the same initial antifungal over any period. Dosage form alterations (eg, intravenous to oral, pill to solution) of azoles were not considered a change in antifungal therapy. However, changes in amphotericin B formulations (deoxycholate to lipid) were considered a change in antifungal therapy.

We assessed median (IQR) all-cause total health care resource utilization and all-cause total costs (2023 US dollars) during index hospitalization and all-cause cumulative total hospitalization and outpatient costs (ie, office visits, emergency room visits, pharmacy, laboratory, pathology, and radiology) at 1, 6, and 12 months after the index date. To be included in 1-, 6-, and 12-month analyses, patients had to have at least that duration of follow-up. To adjust for baseline differences between cohorts, propensity scores were calculated by logistic regression and transformed into weights for stabilized and trimmed inverse probability of treatment weighting (IPTW) adjustment. Variables imbalanced at baseline were used to calculate the propensity score: age, geographic region, payer type, year of index hospitalization, asthma, autoimmune conditions, bacteremia, cancer, chronic obstructive pulmonary disease, pneumonia, immunodeficiencies, myelodysplastic syndrome, neutropenia, transplant history, immunosuppressants, and total 6-month baseline health care costs [11]. Cohorts were deemed well balanced for a given variable if the standardized mean difference between them was <0.10. Chi-square tests and *t* tests or Wilcoxon rank sum tests were used to compare categorical and continuous variables, respectively. *P* < .05 was used to identify statistical significance between cohorts. No adjustments were made for multiple comparisons. Analyses were conducted with SAS version 9.4 (SAS Institute Inc).

## RESULTS

A total of 1192 patients were admitted with IA. Following IPTW, 707 (59.3%) patients changed their initial antifungal therapy and 485 (40.7%) did not. Cohorts were well balanced on baseline characteristics (6 months pre-index) and costs (standardized mean difference <0.1) except corticosteroid use in the 30 days before admission (Table 1). In the change and no-change cohorts, 49.9% and 49.1% of patients were ≥65 years of age, with 38.2% and 39.3% between 45 and 64 years of age. In the change and no-change cohorts, 56.3% and 53.7% of patients were men, 80.8% and 81.3% had commercial or Medicare as their insurance payer, and 86.4% and 86.1% were from the Western and Southern US Census regions. The most prevalent comorbidities were cancer (41.5% and 42.2% in the change and no-change cohorts), chronic obstructive pulmonary disease (34.9% and 34.5%), diabetes (33.2% and 29.2%), and other immunodeficiencies (21.8% and 20.7%). About 1 in 5 patients (21% in both cohorts) had neutropenia during the 6-month pre-index period. Immunosuppressants (18.5% and 17.1%), azoles (38.7% and 36.8%), and corticosteroids within 30 days of IA admission (33.1% and 28.2%) were common pre–index date medications in the change and no-change cohorts. In both cohorts, about 14% and 11% of the population had undergone previous solid organ or bone marrow/hematopoietic stem cell transplant. The median (IQR) time of initial antifungal therapy was shorter in the change vs the no-change cohort (5 [3–10] vs 11 [6–30] days, *P* < .001).

**Table 1.** Post-IPTW Baseline Clinical Characteristics of Patients With Invasive Aspergillosis Who Changed vs Did Not Change Antifungal Therapy

	AFT, No. (%)		
Characteristic^a^	Changed (n = 707)	Did Not Change (n = 485)	SMD^b^
Age, y			0.02
18–44	84 (11.9)	56 (11.5)	
45–64	270 (38.2)	191 (39.3)	
≥65	353 (49.9)	238 (49.1)	
Sex			−0.05
Female	309 (43.7)	225 (46.3)	
Male	398 (56.3)	260 (53.7)	
Geographic region			0.08
Northeast	44 (6.2)	38 (7.9)	
Midwest	52 (7.3)	29 (5.9)	
South	154 (21.8)	106 (21.9)	
West	457 (64.6)	311 (64.2)	
Payer type			0.05
Commercial	254 (36.0)	176 (36.3)	
Medicaid	25 (3.5)	21 (4.2)	
Medicare	317 (44.8)	218 (45.0)	
Cash	0 (0.0)	0 (0.0)	
Unknown	111 (15.7)	70 (14.5)	
Year of index date			0.05
2015	18 (2.5)	12 (2.5)	
2016	65 (9.3)	44 (9.0)	
2017	105 (14.8)	71 (14.7)	
2018	93 (13.1)	57 (11.8)	
2019	121 (17.2)	88 (18.2)	
2020	111 (15.8)	75 (15.4)	
2021	122 (17.2)	87 (18.0)	
2022	72 (10.1)	50 (10.4)	
CCI categories			
0	60 (8.5)	48 (10.0)	−0.05
1	82 (11.6)	53 (10.9)	0.02
2	130 (18.3)	74 (15.3)	0.08
≥3	436 (61.6)	310 (63.9)	−0.05
Comorbidities			
Asthma	88 (12.4)	60 (12.4)	0.00
Autoimmune conditions	48 (6.7)	33 (6.8)	0.00
Bacteremia	40 (5.7)	33 (6.8)	−0.04
Cancer	294 (41.5)	205 (42.2)	−0.01
COPD	246 (34.9)	167 (34.5)	0.01
Diabetes	235 (33.2)	142 (29.2)	0.09
HIV	15 (2.1)	11 (2.2)	0.00
Immunodeficiencies	154 (21.8)	100 (20.7)	0.03
Myelodysplastic syndrome	46 (6.6)	37 (7.5)	−0.04
Neutropenia	148 (21.0)	104 (21.5)	−0.01
Obesity	101 (14.3)	76 (15.7)	−0.04
Pneumonia	333 (47.1)	231 (47.7)	−0.01
Sepsis	166 (23.4)	133 (27.4)	−0.09
Transplant history	172 (24.3)	124 (25.5)	−0.03
SOT	100 (14.2)	69 (14.2)	0.00
BMT/HSCT	76 (10.8)	56 (11.6)	−0.02
Transplant complications	124 (17.5)	87 (17.9)	−0.01
SOT	77 (10.9)	57 (11.8)	−0.03
BMT/HSCT	47 (6.7)	30 (6.1)	0.02
Medications			
Corticosteroids in 30 days prior	234 (33.1)	137 (28.2)	0.11
Immunosuppressants	131 (18.5)	83 (17.1)	0.04
Baseline 6-mo all-cause costs, $ ^c^			−0.02
Mean (SD)	183 656 (335 343)	188 619 (312 936)	
Median (IQR)	69 587 (14 666–220 768)	74 955 (18 044–230 374)	

Abbreviations: AFT, antifungal therapy; BMT, bone marrow transplant; CCI, Charlson comorbidity index; COPD, chronic obstructive pulmonary disease; HSCT, hematopoietic stem cell transplantation; IPTW, inverse probability of treatment weighting; SMD, standardized mean difference; SOT, solid organ transplant.

^a^The following covariates were used to calculate propensity scores for inverse probability of treatment weighting: age group, geographic region, payer type, year of index, asthma, autoimmune conditions, bacteremia, COPD, pneumonia, immunodeficiencies, myelodysplastic syndrome, transplant history, immunosuppressants, cancer, neutropenia, and total healthcare costs.

^b^Cohorts were deemed well balanced for a given variable if the standardized mean difference between cohorts was <0.1.

^c^Inpatient and outpatient costs in the 6 months prior to the index hospitalization for invasive aspergillosis.

The median (IQR) index hospitalization length of stay was 6 days longer in the change vs the no-change cohort (17 [9–31] vs 11 [7–20] days, *P* < .001). There were no differences in the proportion of patients admitted to the intensive care unit during their index stay (70.3% vs 69.4%, *P* = .75), but the change cohort had 6 days longer intensive care unit length of stay than the no-change cohort (15 [8–29) vs 9 [5–17] days, *P* < .001). Total median all-cause index hospitalization costs in the change cohort were significantly higher when compared with the no-change cohort (difference of $65 149, *P* < .001; Table 2). Among hospitalized patients, inpatient pharmacy costs were higher in the change cohort ($22 075 [$7568–$55 852)] vs $11 637 [$4394–$27 393], *P* < .001).

**Table 2.** All-Cause Health Care Utilization and Costs of Treating Invasive Aspergillosis Stratified by Whether Patients Did or Did Not Change Their Index Antifungal Therapy

	Antifungal Therapy, Median (IQR)		
Time Point: Type of Cost	Changed	Did Not Change	*P* Value
Index admission, No.	707	485	
Total cost, $	149 007 (68 013–339 078)	83 858 (45 555–175 068)	<.001
1 mo post-index, No.	590	338	
≥1 rehospitalization, %	19.0	20.1	.67
Total cost, $	199 036 (87 514–443 311)	108 098 (64 999–204 856)	<.001
Hospitalization cost, $	161 472 (70 612–369 485)	91 193 (46 993–167 967)	<.001
Outpatient cost, $	3084 (359–32 055)	4111 (381–33 660)	.78
6 mo post-index, No.	384	205	
≥1 rehospitalization, %	56.8	39.9	<.001
Total cost, $	306 778 (152 167–544 391)	146 958 (80 134–262 352)	<.001
Hospitalization cost, $	195 951 (89 596–402 147)	93 929 (44 375–172 286)	<.001
Outpatient cost, $	48 519 (14 668–126 907	26 895 (9360–69 404)	<.001
12 mo post-index, No.	314	154	
Total cost, $	358 747 (197 194–683 450)	165 794 (83 729–315 345)	<.001
Hospitalization cost, $	215 496 (93 665–468 133)	100 605 (44 353–171 210)	<.001
Outpatient cost, $	78 285 (28 102–173 203)	43 164 (13 898–95 697)	<.001

Over 12 months of follow-up from the index date, the median (IQR) number of emergency department visits per person per year was 1 (0–4) in the change cohort and 0 (0–4) in the no-change cohort (*P* = .35). At 1 month after the index date, no difference in the proportion of patients with ≥1 all-cause rehospitalization was observed (*P* = .67); however, median all-cause total inpatient and outpatient costs were higher in the change vs the no-change cohort (difference of $90 938, *P* < .001), driven primarily by hospitalization costs (difference of $70 279, *P* < .001). At 6 months post–index date, the proportion of patients with ≥1 all-cause rehospitalization was higher in the change cohort (56.8% vs 39.9%, *P* < .001). Treating the change cohort was again more costly (difference of $159 820, *P* < .001), driven by hospitalization and outpatient costs (differences of $102 022 and $21 624, *P* < .001 for both). Cost at 12 months post–index date followed a similar pattern, with the change cohort costing $192 953 (*P* < .001) more than the no-change cohort, with increases in hospitalization ($114 891) and outpatient ($35 121) costs (*P* < .001 for both).

## DISCUSSION

In the present study, nearly 60% of admitted patients with IA changed their initial antifungal therapy, with many doing so within 1 to 2 weeks of treatment initiation. These findings are supported by a study that Henao-Martinez et al performed in the TrinetX global research network database, which also showed changing antifungal therapy occurring often and early during the management of IA [8]. We further demonstrated a statistically significant $65 149 higher median index inpatient cost in the antifungal therapy change vs no-change cohort. Excess all-cause treatment costs in the change cohort were also statistically and persistently higher than in the no-change cohort over the next year of follow-up, with the difference in costs exceeding $190 000 at 12 months and driven by excesses in inpatient and outpatient costs. To our knowledge, this is the first published study to evaluate the association between changing initial IA antifungal therapy and index hospitalization and subsequent-year all-cause healthcare costs. Based on our findings, it would appear that interventions to maximize the possibility of optimal initial antifungal therapy are vital to the efficient use of health care resources and the economic outcomes of patients with IA.

Our study has limitations worth consideration. First, as a claims database study, misclassification bias is a concern [12]. IA was presumed by *ICD-10-CM* diagnosis codes, and neither a formal diagnosis nor the severity of IA could be verified due to the lack of available laboratory data. Our study may also be prone to immortal time bias. Second, the reasons for change in antifungal therapy were not identifiable. Multiple factors may influence the decision to change antifungal therapy [4, 7]: the development of a new infection, recognition of a resistant pathogen, clinical deterioration or progression of the original lesion, safety or tolerability concerns, drug-drug interactions, or financial constraints [7, 8]. Third, while ours was a modestly sized study, its sample size did not permit analyses of health care resource utilization or costs specifically due to IA. Fourth, despite our use of IPTW methods, residual confounding can never be fully ruled out in nonrandomized studies [11]. Next, health care resource utilization could have occurred outside of IQVIA's data partners. Such utilization would not be captured and could result in an underestimation of resource usage and costs. Last, because our study spanned a long time frame (2015–2022), it is unclear how changes in the practice for managing IA, such as approval of new antifungals, improved diagnostics, and updated treatment guidelines, may have influenced our results.

## CONCLUSIONS

In this contemporary cohort of patients with IA, most patients changed their antifungal therapy, with many doing so within 1 to 2 weeks of treatment initiation. Changing antifungals was associated with significantly longer hospital and intensive care unit lengths of stay and higher inpatient costs. Future studies involving larger populations are warranted to better understand the cost of treating IA and to identify reasons for change in initial antifungal therapy. Such studies will be key to identifying and addressing unmet needs for IA management, including improved diagnostics and novel oral antifungals with activity against resistant pathogens that have fewer toxicities, adverse events, and drug interactions.

## Notes

***Author contributions.*** Conceptualization: B. L., C. I. C., V. R. A., L. P., M. D. Data analysis: V. R. A., L. P., M. D. Data interpretation: all authors. Writing (original draft preparation): B. D. A., M. J., C. I. C., B. L. Writing (review and editing): all authors. Study supervision: B. L.

***Financial support.*** F2G, Inc.
