# Correction to: Association Between the Need to Change Initial Antifungal Therapy and Treatment Costs in Patients With Invasive Aspergillosis

**DOI:** 10.1093/ofid/ofaf161

**Published:** 2025-03-18

This is a correction to: Barbara D Alexander, Melissa Johnson, Mark Bresnik, Vamshi Ruthwik Anupindi, Lia Pizzicato, Mitchell DeKoven, Belinda Lovelace, Craig I Coleman, Association Between the Need to Change Initial Antifungal Therapy and Treatment Costs in Patients With Invasive Aspergillosis, *Open Forum Infectious Diseases*, Volume 12, Issue 1, January 2025, ofae747, https://doi.org/10.1093/ofid/ofae747

In the originally published version of the manuscript there was an error in Table 2. In Table 2, the median total cost (in $) value for patients who changed antifungal therapy is emended to read: “149 007” instead of: “14 900”.

